# Recent advances in bedside microcirculation assessment in critically ill patients

**DOI:** 10.5935/0103-507X.20170033

**Published:** 2017

**Authors:** Philipe Franco do Amaral Tafner, Felipe Ko Chen, Roberto Rabello Filho, Thiago Domingos Corrêa, Renato Carneiro de Freitas Chaves, Ary Serpa Neto

**Affiliations:** 1 Faculdade de Medicina do ABC - Santo André (SP), Brasil.; 2 Unidade de Terapia Intensiva Adulto, Hospital Israelita Albert Einstein - São Paulo (SP), Brasil.

**Keywords:** Shock, Septic shock, Hemodynamics, Resuscitation, Microcirculation, Microscopy, video

## Abstract

Parameters related to macrocirculation, such as the mean arterial pressure, central venous pressure, cardiac output, mixed venous saturation and central oxygen saturation, are commonly used in the hemodynamic assessment of critically ill patients. However, several studies have shown that there is a dissociation between these parameters and the state of microcirculation in this group of patients. Techniques that allow direct viewing of the microcirculation are not completely disseminated, nor are they incorporated into the clinical management of patients in shock. The numerous techniques developed for microcirculation assessment include clinical assessment (e.g., peripheral perfusion index and temperature gradient), laser Doppler flowmetry, tissue oxygen assessment electrodes, videomicroscopy (orthogonal polarization spectral imaging, sidestream dark field imaging or incident dark field illumination) and near infrared spectroscopy. In the near future, the monitoring and optimization of tissue perfusion by direct viewing and microcirculation assessment may become a goal to be achieved in the hemodynamic resuscitation of critically ill patients.

## Abstract (pt)

Parâmetros relacionados à macrocirculação, como pressão arterial média, pressão venosa central, débito cardíaco e saturação venosa mista e central de oxigênio, são comumente utilizados na avaliação hemodinâmica de pacientes graves. No entanto, diversos estudos demonstram que existe dissociação entre estes parâmetros e o estado da microcirculação neste grupo de pacientes. Técnicas que permitem a visualização direta da microcirculação não estão completamente difundidas e nem incorporadas ao manejo clínico dos pacientes em choque. Entre as inúmeras técnicas desenvolvidas para avaliação da microcirculação encontram-se: avaliação clínica (por exemplo: índice de perfusão periférica e gradiente de temperatura); fluxometria por laser Doppler; eletrodos de avaliação de oxigênio tecidual; videomicroscopia (imagem espectral por polarização ortogonal, análise em campo escuro de fluxo lateral, ou iluminação incidental em campo escuro); e espectroscopia no infravermelho próximo. A monitorização e a otimização da perfusão tecidual por meio da visualização direta e da avaliação da microcirculação pode, em um futuro próximo, tornar-se uma meta a ser atingida na ressuscitação hemodinâmica dos pacientes graves.

## INTRODUCTION

**Figure 1:**
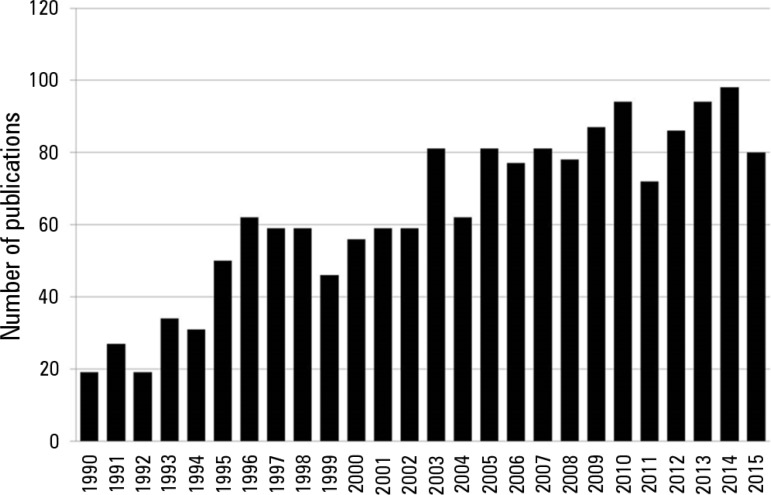
Number of publications on microcirculation in recent years. Search terms used: (Blood Circulation [mh] OR Microcirculation [mh] OR Microvascular Network [tiab] OR Microvessels [mh]) AND ("ICU" OR "critically ill" OR "intensive care unit"). There were no restrictions regarding the study design and age of included participants.

Parameters related to macrocirculation, such as the mean arterial pressure (MAP), central venous pressure (CVP), cardiac output (CO), mixed venous saturation (SvO_2_) and central venous oxygen saturation (ScvO_2_), are commonly used in the hemodynamic assessment of critically ill patients.^(1-4)^ However, several studies have shown that there is a dissociation between these parameters and the microcirculation state in this group of patients.^(5-7)^ The recent development of new techniques for microcirculation assessment, coupled with the growing number of studies published in this area (Figure 1), has helped in understanding the microcirculation's characteristics,^(8)^ especially its physiopathology in different states of shock.^(9,10)^

It is postulated that changes in microcirculatory blood flow may be directly related to the development of organic dysfunctions.^(5-7,11)^ In addition, the persistence of microcirculatory changes, despite macro-hemodynamic optimization, is associated with higher mortality.^(6,12)^ Therefore, it is suggested that the assessment and consequent early optimization of microcirculatory parameters may be associated with better outcomes in critically ill patients.^(8)^

Arterial lactate and ScvO_2_ are parameters frequently used as targets in the treatment of septic shock, but they are considered global tissue perfusion parameters and do not reflect blood flow in different regions.^(13,14)^ Furthermore, such markers do not represent a direct assessment of the microcirculation function since there is no viewing or structural analysis of the same.^(8-10)^

Despite the relevance of the subject in terms of recent research, there are relatively few reviews addressing recent advances in microcirculation assessment and its bedside use in critically ill patients. Thus, the purpose of the present review was to describe the structure and functions of microcirculation, its changes in physiological and pathological conditions, and the different methods currently available for its assessment in the critically ill patient.

## MICROCIRCULATION

### Characteristics of microcirculation in physiological conditions

**Figure 2:**
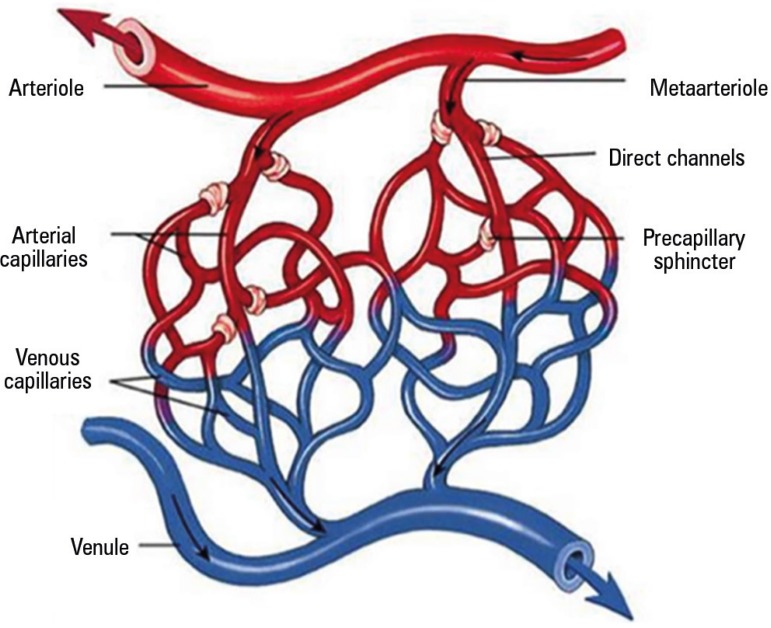
Microcirculation anatomy.

The microcirculation consists of vessels with diameters of less than 100 µm, including arterioles, metarterioles, capillaries and venules (Figure 2).^(15)^ Arterioles are responsible for maintaining the vascular tonus and, consequently, for control of the pressure gradient between the proximal and distal capillaries.^(16)^ In this manner, they promote local blood flow control, according to the tissue's metabolic demand.^(9,10)^

The capillaries originate from the arterioles and are lined by a single layer of endothelial cells. They are responsible for the exchange of oxygen and nutrients between the intravascular and adjacent cells.^(16)^ In resting conditions, only 20 to 30% of the capillaries are "functioning", that is, actively participating in tissue perfusion.^(8,9)^ In tissue hypoxia, capillary recruitment occurs rapidly due to the opening of precapillary sphincters.^(7,8)^ This recruitment allows for the maintenance of a dynamic environment for gas exchange and supplies peripheral blood nutrients to the tissue.^(17,18)^ Furthermore, the capillary network architecture and its vascular density vary according to the functions performed by the various organs, even acting as a counter-mechanism in some organs.^(17,18)^ The venules, in turn, play an important role in the immune response and, due to their degree of distensibility and high capacitance, also allow storage and mobilization of large amounts of blood.^(9,19,20)^

**Figure 3:**
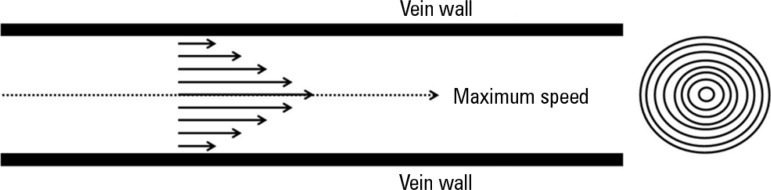
Poiseuille's Law. Flow rate according to vessel radius (left) and the hypothetical concentric rings within a blood vessel (right).

The microcirculation should be understood as a functional distribution system of blood flow and thus of oxygen and nutrients to cells and tissue. Poiseuille's law demonstrates that if one observes the concentric rings within the vessels, by virtue of laminar flow, one can see that the flow velocity of each ring is different; thus, the blood near the ring wall has a lower flow rate than the more central blood flow, mainly due to adherence of the formed blood components to the vascular endothelium (Figure 3).^(21,22)^ By factoring in the different speeds of all of the concentric blood flow rings and multiplying them by their respective areas, the following formula is obtained, known as Poiseuille's law:

$$F=\pi \Delta {\mathrm{Pr}}^{4}\;/\;\left(8\;\eta \;L\right)$$

where F is blood flow; ΔP, the pressure difference between the ends of the vessel; r, the radius of the vessel; L, its length; and *η*, blood viscosity.^(22)^

The rheological properties of fluids (viscosity is the best known of them) are extremely important to the maintenance of blood flow in microcirculation.^(23-25)^ In general, blood rheology is responsible for the amount of movement and affects not only the flow pattern but also the functional capillary density.^(23)^ Vascular resistance, controlled by the endothelium, can also change regional blood flow dramatically.^(23,24)^

In addition to providing the tissue with oxygen by its diffusion in the arterioles and capillaries, hemoglobin has characteristics that are important to the microcirculation, such as its direct effect on the maximum distance between the place of diffusion and the mitochondria; the formation of its molecules, which have two different forms designated taut and relaxed;^(24)^ and vasoactive control of the release of substances, such as adenosine triphosphate (ATP) and nitric oxide (NO) derivatives.^(8,21,24,25)^

Finally, the convective and diffusive components of microcirculatory blood flow are essential to the transport of oxygen to the tissue.^(26)^ The convective component is directly related to blood flow in the microcirculation, being essentially determined by the number of erythrocytes and the saturation thereof.^(26)^ The diffusive component, in turn, is directly related to the difference between the partial oxygen pressure (PO_2_) levels in the capillaries and mitochondria, the oxygen diffusion distance and the gas exchange surface.^(26)^

### Microcirculation in pathological conditions

Most publications on microcirculation dysfunction address patients with septic shock. It is postulated that changes in the microcirculation reduce the supply of oxygen to the mitochondria, impairing ATP production.^(27)^ Changes in the microcirculation in sepsis occur due to inflammation, activation of coagulation and the complement system and damage to the capillary endothelium.^(28)^

Vessel tonicity is regulated by the endothelial cells,^(28)^ which produce vasoactive molecules responsible for regulating arteriolar contraction and help with blood pressure control.^(28)^ These molecules include vasodilating substances, such as NO and prostacyclin, and vasoconstricting substances, such as thromboxane A2, endothelin and platelet activating factor (PAF).^(28,29)^ Critically ill patients may present imbalances between these components, causing vasomotor instability and regional hypoperfusion.^(28,29)^

One of the major changes in the endothelium during sepsis is an increase in its permeability, or the loss of barrier function, which leads to an imbalance in the circulation of blood elements and tissue edema.^(28)^ Hemoglobin also has crucial importance in this context.^(29)^ In an experimental sepsis model, a significant reduction in erythrocyte deformability, contributing to microcirculatory dysfunction, has been demonstrated.^(29)^ Moreover, patients with sepsis suffer impairments of the convective and diffusive components of microcirculatory blood flow, resulting in a heterogeneous and insufficient tissue oxygen supply.^(27)^

The relationship between systemic and regional perfusion closely depends on the cause of circulatory shock.^(30-33)^ In cardiogenic shock, for example, all microcirculatory variables undergo change, such as reductions in the diameter of arterioles and the functional capillary density.^(30-33)^ In patients with heart failure, intravenous infusion of nitroglycerin has been able to increase functional capillary density, even with a reduction in cardiac filling pressures, demonstrating the independence of the microcirculation in relation to macro-hemodynamic variables and their dynamic character.^(31,32)^

In hemorrhagic shock, microcirculatory changes are early and may reflect a state of tissue perfusion with lower oxygen consumption.^(33)^ In an experimental model in pigs, it has been demonstrated that, with the removal of 35% of the blood volume, rapid decreases in the cardiac index, SvO_2_ and oxygen delivery (DO_2_) occurred, along with an increase in lactate and a reduction in tissue oxygen saturation (StO_2_) in skeletal muscle.^(34)^ Only animals that received aggressive volemic resuscitation showed an increase in StO_2_ values, demonstrating how this noninvasive microcirculation measure may be relevant at the bedside.^(34)^ A study conducted on patients admitted to the intensive care unit (ICU) for hemorrhagic shock demonstrated that, even in the presence of normal macrocirculatory parameters, the sublingual microcirculation was dysfunctional for up to 3 days post-shock.^(35)^ Furthermore, the microcirculatory indices assessed in the study changed in all trauma patients, but these changes were more pronounced in those patients with hemorrhagic shock.^(35)^

## MICROCIRCULATION ASSESSMENT

By definition, any equipment that analyzes the microcirculation can do so in only the vascular bed being assessed. However, it may be considered that the area being investigated is a window that reflects changes that are likely to be observed elsewhere.^(8)^ Among the many techniques developed to assess microcirculation are clinical assessment (peripheral perfusion index and temperature gradient, among others), laser Doppler flowmetry, tissue oxygen assessment electrodes (PO_2_), videomicroscopy (orthogonal polarization spectral imaging (OPS), sidestream dark field (SDF) or incident dark field illumination (IDF))^(36)^ and near infrared spectroscopy (NIRS).^(8)^

Microcirculation assessment can be performed on different types of tissue, according to the technique and apparatus used. The sublingual area is often used to perform videomicroscopy, as it is easily accessible and noninvasive and is potentially reliable in terms of patient monitoring and management.^(8)^ Furthermore, studies suggest that partial sublingual carbon dioxide pressure (PslCO_2_) is directly related to partial gastric carbon dioxide pressure (PgCO_2_), indicating that the sublingual region is a good indicator for the indirect assessment of splanchnic microcirculation.^(37)^ It is important to note that microcirculation assessment is currently restricted to research protocols, and its use at the bedside as a therapeutic goal also depends on greater scientific development in this area.^(37,38)^ Table 1 presents a brief summary of the main methods of microcirculation analysis.

**Table 1:** Main microcirculation assessment techniques

Technique	Principles	Application	Limitations	Measured or calculated variables	Authors
Laser Doppler flowmetry	Laser Doppler flow analysis	Microcirculatory functional integrity assessment	Does not distinguish between blood flow in the arterioles, capillaries and venules	Relative blood flow Hemoglobin content	De Backer et al.^(8)^ and Micheels et al.^(39)^
Videomicroscopy	Emission of polarized light that, when absorbed, produces an image representing the RBCs as black bodies Available technologies: OPS, SDF and IDF	Direct viewing of microcirculation	Microcirculation analysis limited to the assessed window Image analysis performed offline Image acquisition affected by operator skill	Total vascular density Functional capillary density Proportion of perfused vessels Proportion of small perfused vessels Flow heterogeneity index	Aykut et al.,^(36)^ De Backer et al.,^(40)^ Boerma et al.^(41)^ and Carsetti et al.^(42)^
PO_2_ assessment electrodes	Transcutaneous electrode with sensor that detects oxygen and carbon dioxide by means of electrical and chemical reactions	Tissue flow adequacy in low-flow situations	Pulmonary dysfunction	Transcutaneous oxygen pressure Transcutaneous carbon dioxide pressure	Vesterager,^(43)^ and Lima^(44)^
NIRS	Near infrared application with several wavelengths Molecular components of different types of tissue have different absorption and light dispersion characteristics	Noninvasive and continuous peripheral tissue oxygenation monitoring	Adipose tissue thickness or bone width at NIRS application site Myoglobin effect on tissue oxygenation measurement Interstitial edema effect on NIRS signal	StO_2_ Total hemoglobin VOT-derived variables (deoxygenation and reoxygenation speed)	Lima et al.^(45)^

NIRS - near infrared spectroscopy; StO_2_ - tissue oxygen saturation; VOT - vascular occlusion test; OPS - orthogonal polarization spectral imaging; SDF- sidestream dark field; IDF - incident dark field illumination.

### Clinical assessment

During circulatory failure, the vital organs demonstrate vasomotor self-regulation whereby they are able to maintain blood flow, despite the presence of hypotension.^(22)^ However, cutaneous circulation has no such self-regulation, resulting in a decrease in skin perfusion and the consequent fall of regional temperature secondary to vasoconstriction.^(46)^

This skin temperature drop can be assessed via peripheral-ambient (dTp-a) and central-peripheral (dTc-p) temperature gradients. Assuming that the ambient temperature remains constant, the dTp-a gradient decreases while dTc-p increases during situations of circulatory collapse.^(46,47)^ Under physiological conditions, dTc-p shows variations between 3 - 7°C.^(47)^

Several studies have been conducted to assess the relationship between the temperature gradient and vasoconstriction or vasodilation secondary to local blood flow changes.^(47,48)^ One study examined the blood flow and temperature gradients in the forearm and fingertip in volunteers subjected to an artificial vasodilation and vasoconstriction process.^(47)^ The principal findings revealed that differences of only 1.5°C were detected in vasoconstriction situations.^(47)^ Thus, peripheral temperature gradient analysis has demonstrated great value in terms of vasoconstriction and vasodilation, as a strong correlation between dTp-a and serum lactate levels has been demonstrated.^(49)^

Another index that may be used at the bedside to assess circulatory failure situations is the peripheral perfusion rate.^(50)^ This method uses pulse oximetry and is able to distinguish between the pulsatile (blood) and the non-pulsatile (other tissue) components and between hemoglobin and oxygenated hemoglobin.^(50)^ An important point is that calculation of the index is performed independently of the oxygen saturation value.^(46,50)^ Peripheral perfusion index values of less than or equal to 1.4 have been related to the presence of tissue hypoperfusion.^(46)^

Capillary refill time is useful in identifying blood hypoperfusion states in hemodynamically unstable patients.^(51)^ It is measured by applying firm pressure to the distal phalanx of the right and left index fingers for 15 seconds each.^(51)^ The time in seconds to return to normal skin color is determined using a stopwatch.^(51)^ A time of 5 seconds is set as the upper normal limit for this test, but this rate varies according to age and gender.^(46,51)^ The capillary refill time may be up to 2.9 seconds in healthy women and up to 4.5 seconds in the elderly.^(46,51)^ Many studies suggest that the correlation between the capillary refill time and blood pressure or CO is not reliable and is a good predictor of only dehydration, reduced systolic volume and increased serum lactate in children.^(46)^

In the ICU, a skin assessment looking for clinical signs that may correlate with tissue hypoperfusion is the usual practice. Mottling constitutes a change in skin color, and its pathophysiology is not completely clear.^(52)^ However, it is postulated that such changes result from skin hypoperfusion. The mottling score consists of a semi-quantitative assessment of skin mottling, based primarily on the extent of its presence in the assessed area (usually the knee region).^(52)^ The mottling score is easily applied at the bedside, has good correlation with tissue perfusion variables, such as lactate and urine output, and has good predictive value when assessing mortality in patients with septic shock.^(52)^

### Laser Doppler flowmetry

Laser Doppler flowmetry analyzes the relative microcirculatory blood flow and reserve by means of microvascular reactivity testing.^(39)^ For the measured flow to represent the average flow of at least 50 vessels, including arterioles, capillaries and venules of various sizes, the sample volume of the current laser Doppler apparatus should be between 0.5 and 1 mm^3^. The method uses a confocal technique to measure vascular density and diameter and blood flow.^(8,39)^ In the microvascular reactivity test, the upward slope after the occlusion is a marker of endothelial reactivity and blood rheology and can be used as a functional microvascularization integrity parameter.^(8)^ The measurement can be performed on any area of intact skin.^(39)^

### Partial oxygen pressure assessment electrodes

The potential uses of electrodes for PO_2_ assessment include the accurate assessment of tissue PO_2_.^(43,53)^ The sample volume analyzed by these electrodes comprises at least one hundred microvessels, including arterioles, capillaries, venules, interstitial and other cells, all of which contribute to the final assessed PO_2_ value.^(43,53)^ Their main use is in the indirect assessment, via PO_2_ levels, of perfusion and/or regional oxygenation, especially in low-flow conditions.^(8,43,53)^ One of the main problems with the use of electrodes is the fact that it is impossible to assess microvascular perfusion directly.

Recent advances have made it possible to measure PO_2_ and carbon dioxide continuously and non-invasively using transcutaneous sensors.^(44)^ Carbon dioxide is approximately 20 times more diffusible than oxygen, and transcutaneous oxygen measurement (PtCO_2_) is more sensitive to changes in perfusion than transcutaneous carbon dioxide measurement.^(44)^ The oxygen challenge test involves temporarily increasing the inspired oxygen fraction (FiO_2_) used and monitoring the PtcO_2_ response. In patients with normal lung function, increased FiO_2_ is associated with a parallel increase in PtcO_2_ since, in patients with adequate blood flow, the PtcO_2_ and PaO_2_ values are almost identical.^(44)^ A lack of increase in PtcO_2_ after an increase in FiO_2_ suggests probable perfusion dysfunction and portends a worse outcome in septic shock patients.^(44)^

### Videomicroscopy

**Figure 4:**
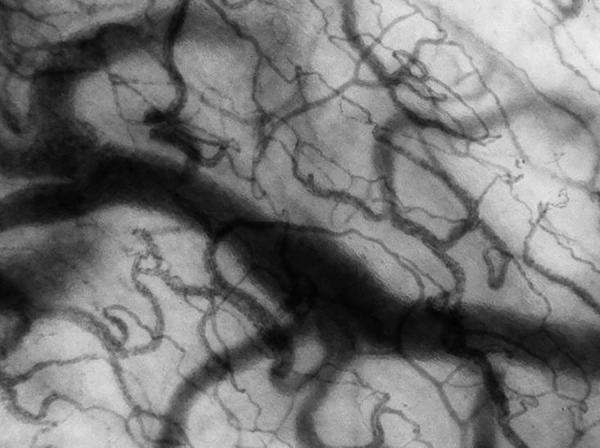
Example of a microcirculation image obtained via videomicroscopy.

Videomicroscopy assesses microcirculation directly by emitting polarized green light, which, when absorbed, produces an image representing the red blood cells (RBCs) as black bodies.^(40,41)^ This technique can be applied to organs that have a thin epithelial layer, such as the sublingual region, in which capillaries and venules of various sizes can be observed.^(41)^ The techniques used in microcirculation videomicroscopic assessment are OPS and SDF analyses - the latter being the most used in recent years.^(40)^ An example of a microcirculatory image obtained by videomicroscopy is shown in figure 4. Importantly, the analysis of images obtained using these methods is performed offline. Therefore, the impossibility of achieving automatic microcirculation analysis does not allow clinical decision making at the bedside and limits the use of these techniques to research protocols.

This situation may change due to the recent development of a new IDF technique, described as third-generation videomicroscopy.^(36)^ Cytocam-IDF, the only equipment able to perform this type of analysis to date, consists of a probe that incorporates IDF illumination with an array of high-resolution image projector lenses.^(36)^ These pictures are projected onto a high-density sensor controlled by a computer synchronized with an illuminated unit. A recent study comparing the results of this device with SDF showed that Cytocam-IDF could detect more capillaries (30% more) and generate better quality images than the SDF technique.^(36)^ Similar results were obtained in different preliminary validation studies involving neonates.^(54)^

There are five steps during assessment that are essential to the correct use of videomicroscopy: the assessment of five sites per organ, the avoidance of pressure artifacts, the removal of secretions prior to assessment, the use of proper focus and the calibration of contrast and recording quality.^(40,55)^

The main functional features of the microcirculation analyzed by videomicroscopy include vascular density (responsible for oxygen supply by diffusion), the pattern and intensity of microcirculatory blood flow (responsible for oxygen supply by convection) and flow heterogeneity (distributional changes and shuntings).^(40)^ Several scores have been developed for analysis of the results obtained by videomicroscopy.

Generally, a form with three horizontal rows and three vertical lines is placed in front of the screen while each video sequence is played.^(5)^ Vascular density is calculated as the number of vessels crossing these lines, divided by the total line length. The total vascular density corresponds to the total number of vessels (both small and large, with and without normal flow), while the functional capillary density is the number of well-perfused small vessels (< 20 µm) per unit area.^(5)^ Flow type is defined as continuous, intermittent or absent.^(5)^ The vessels are usually separated into large (mainly venules) and small (mainly capillaries), using a cutoff value of 20µm in diameter.^(5)^ Vessel perfusion (total, large and small) is defined as the proportion of perfused vessels (PPV), calculated as the number of continuously perfused vessels during an observation of 20 seconds, divided by the total number of vessels of the same type.^(5)^ Thus, the proportion of small perfused vessels corresponds to the proportion of well-perfused vessels with diameters < 20 µm (mainly capillaries).^(5)^ The flow heterogeneity index is defined as the difference between the maximum and minimum PPV proportion assessed at each point of five areas, divided by its own mean value.^(5)^ Each of these microcirculatory parameters is obtained from an average of five video streams or possibly more.

Additionally, a second form, having vertical and horizontal lines, can also be placed in front of the screen to separate the image into four quadrants.^(7,36,42,55)^ In this image, microvascular flow is characterized as absent (0), intermittent (1) slow (2) or normal (3).^(7,8,36,42,55)^ The mean value of these four quadrants is reported as the microvascular flow index.

Trzeciak et al. described a way of assessing microcirculation based on the microvascular flow index.^(7)^ This method proposes to divide the obtained image into four quadrants and to determine which of these quadrants has the predominant flow type, classified as described above.^(7)^ In addition, the authors added a heterogeneity index, which can be obtained by subtracting the area of the greatest flow rate from the area with the lowest flow rate and then dividing by the mean flow velocity of all assessed areas.^(7)^ Normal microcirculation exhibits minimal blood flow heterogeneity,^(56)^ and there must be an adequate relationship between perfusion and metabolism (or the supply and demand of oxygen and nutrients) to prevent hypoxia-induced cell damage.^(57)^

Generally, tissue is better able to adapt to low-flow situations with homogeneous microcirculation than in heterogeneous flow situations.^(57,58)^ By reducing functional capillary density and creating a heterogeneous flow, oxygen diffusion distance increases, and as a result, poor tissue oxygen extraction is observed.^(8,40)^ Thus, assessment of the microcirculation is of great value, as it identifies poor peripheral perfusion conditions, even in situations with normal or increased SvO_2_.

Recently, De Backer et al. stated that the result of videomicroscopic microcirculation assessment must always show the density of perfused vessels (as an estimate of functional capillary density), the PPV and the microvascular flow index for all vessels, large and small, along with the heterogeneity index.^(40)^

### Near infrared spectroscopy

NIRS is a technique that measures the chromophores (parts or groups of atoms responsible for the color of a molecule) of oxyhemoglobin, deoxyhemoglobin, myoglobin and cytochrome aa3 in any given tissue.^(8)^ By measuring the oxy- and deoxyhemoglobin fractions, one can calculate StO_2_, the total tissue hemoglobin (THb) and the absolute tissue hemoglobin index (THI); THb and THI are two microcirculatory blood volume indicators.^(59,60)^ The measurements made using NIRS may be affected by the amount of adipose tissue and by the presence of edema at the assessment site.^(59,60)^ The thenar eminence region has been the most used because of the thickness of skin and because the adipose tissue covering this muscle is less affected by body weight variations.^(59,60)^

NIRS does not measure blood flow directly, complicating the interpretation of tissue oxygenation by means of absolute StO_2_ levels.^(61)^ The analysis of changes in StO_2_ during a brief period of ischemia on the forearm, known as the vascular occlusion test (VOT), provides a dynamic assessment of microvascular reserve in just a few minutes.^(45,61)^ Arterial and venous vascular occlusion can be achieved when inflating a sphygmomanometer positioned on the patient's arm, above the systolic blood pressure, with the aim of inducing ischemia in the thenar muscle and causing changes in StO_2_. There is still no consensus regarding the intensity and duration of VOT; two strategies have been described: the use of VOT based on inflation time, because the maximum ischemic vascular response is obtained within a few minutes,^(62)^ and the use of VOT based on a drop in StO_2_, seeking a 40% StO_2_ target to minimize inter-individual variations in the VOT response (Figure 4).^(61,63)^

The desaturation rate (R_des_, %/seconds) in the thenar muscle, after vascular obstruction, can be used to estimate this muscle's oxygen consumption.^(61-64)^ The product of the absolute value of R_des_ and the mean THI value quantifies the amount of hemoglobin desaturated in the tissue. After deflation of the sphygmomanometer, there is rapid restoration of blood flow, called the resaturation rate (R_res_, %/seconds).^(61-64)^ During this reactive hyperemia, StO_2_ can reach higher levels than baseline StO_2_, indicating post-ischemic vasodilation and capillary recruitment.^(61-64)^

The main limitations of this method include the fact that NIRS does not directly assess microcirculatory flow and globally checks a combination of arterioles, capillaries and venules. Furthermore, the NIRS signal is limited to vessels with diameters of less than 1 mm.^(58,59)^

### Potential therapeutic applications of the use of microcirculation

The emergence of techniques that allow direct viewing of the microcirculation have led to studies that are focused on interventions modifying the microcirculation of critically ill patients. The main interventions studied include the use of vasodilators to obtain better microcirculatory flow homogeneity. In patients with acute heart failure, the use of low nitroglycerin doses has resulted in an increase in functional capillary density.^(32)^ In patients with severe sepsis or septic shock, various interventions have demonstrated potential effects on the microvasculature after adequate resuscitation: (1) the use of nitroglycerin was associated with increased microcirculatory blood flow;^(56)^ (2) dobutamine infusion caused significant increases in vascular density and capillary perfusion;^(65)^ and (3) the infusion of Ringer's lactate or 4% albumin solution also increased small vessel density and perfusion.^(66)^ In contrast, RBC transfusion in a patient with severe sepsis had no significant effect on the microcirculation.^(67)^ It is important to highlight the role of individual variation in RBC transfusion. Possible causes of the lack of a transfusion effect may include changes in rheological properties, loss of RBC deformability and reduced 2,3-diphosphoglycerate concentrations.^(67)^ Furthermore, RBC storage time also showed no relationship with potential microcirculatory changes.^(67)^ Finally, in patients with septic shock, norepinephrine infusion to raise MAP values above 65mmHg did not cause changes to the sublingual microcirculation pattern and did not lead to any improvements in parameters usually adopted in tissue perfusion monitoring, such as arterial lactate, anion gap and the difference between the partial carbon dioxide pressure of the gastric mucosa and the partial pressure of arterial carbon dioxide.^(68)^

## CONCLUSION

The isolated assessment of macro-hemodynamic parameters and global perfusion markers as shock treatment goals does not seem to be entirely appropriate, as these parameters do not assess the state of tissue microcirculation in these patients. However, techniques that allow viewing and assessment of the microcirculation are not yet fully developed and incorporated into clinical practice. Therefore, advances in this area are imperative, given that the monitoring and optimization of tissue perfusion by direct viewing and microcirculation management may become an achievable goal in the near future in the hemodynamic resuscitation of critically ill patients.

## Figures and Tables

**Figure 5:**
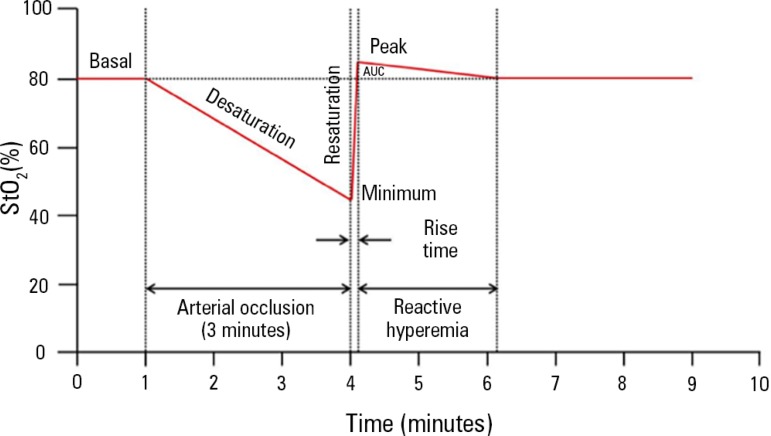
Image of a vascular occlusion test monitored by near infrared spectroscopy StO_2_ - tissue oxygen saturation; AUC- area under the curve.
